# Neutrophil-to-lymphocyte ratio predicts nodal involvement in unfavourable, clinically nonmetastatic prostate cancer patients and overall survival in pN1 patients

**DOI:** 10.1038/s41598-023-27542-2

**Published:** 2023-01-09

**Authors:** Piotr Zapała, Karolina Garbas, Zbigniew Lewandowski, Aleksander Ślusarczyk, Cezary Ślusarczyk, Łukasz Mielczarek, Giancarlo Marra, Benjamin Pradere, Pawel Rajwa, Łukasz Zapała, Piotr Radziszewski

**Affiliations:** 1 Clinic of General, Oncological and Functional Urology, Medical University of Warsaw, Lindleya 4, 02-005 Warsaw, Poland; 2 Department of Epidemiology, Medical University of Warsaw, Warsaw, Poland; 3 Second Department of Urology, Centre of Postgraduate Medical Education, Warsaw, Poland; 4 Division of Urology, Department of Surgical Sciences, San Giovanni Battista Hospital, University of Studies of Torino, Turin, Italy; 5 Department of Urology, Medical University of Vienna, Vienna, Austria; 6 Department of Urology, Medical University of Silesia, Zabrze, Poland

**Keywords:** Urology, Prostate

## Abstract

The aim of our study was to determine the clinical utility of neutrophil-to-lymphocyte ratio (NLR) in predicting presence and prognosis of nodal involvement in patients treated with radical prostatectomy (RP) due to prostate cancer. This single-centre retrospective study included 205 patients treated with RP and lymphadenectomy between 2012 and 2018. Logistic regression and Kaplan–Meier analyses were performed to evaluate the prognostic value of preoperative NLR in terms of nodal spread and survival. Patients staged pN1 presented lower mean NLR (2.53 vs 3.86; *p* = 0.0025) compared to pN0 patients. On multivariable analysis of different haematological markers, only NLR exceeding the median (≥ 2.7) predicted pN1 (OR = 0.38; *p* = 0.0367) independently of biopsy grading and PSA. In internal validation (n = 31 pN1, n = 174 pN0) on the bootstrapped dataset using a spare cutoff of NLR ≥ 4.1 would allow sparing lymphadenectomy in 22.09% pN0 patients, missing 6.45% pN1 (NPV 92.66%; 95% CI 84.91–100%). Noticeably, in pN1 patients NLR ≥ 2.7 correlated with shorter overall survival (*p* = 0.0196), despite its association with reduced risk of pN1. High pre-prostatectomy NLR was negatively associated with pN1, yielding high NPV in internal validation. Simultaneously, high NLR in pN1 patients was associated with shorter survival.

## Introduction

Although extended pelvic lymphadenectomy (eLND) performed during radical prostatectomy has not been associated with improved oncological outcomes, eLND remains the gold standard of nodal staging and provides crucial prognostic information that can drive decisions on further treatment of prostate cancer (PCa)^1^. On the other side, eLND increases the morbidity of the surgery with overall complication rates exceeding 20%^1^. To identify candidates for eLND multiple nomograms have been introduced and validated^2,3^, however, this only partially addresses the issue of eLND overuse in N0. Due to the following reasons, preoperative markers of nodal involvement (NI) are being constantly investigated.

Since in most cases nodal involvement will lead to biochemical recurrence (BCR) which might in turn compromise cancer-specific survival (CSS)^4^, the majority of patients staged pathologically as N1 (pN1) will require androgen deprivation therapy (ADT) and at least a third will receive radiotherapy to prevent progression^5^. Simultaneously, along with the increasing availability of PET-PSMA, patients presenting as radiologically suspected of bearing N1 (cN1 M0) have become the focus of attention^6^. Although cN1 patients are recommended to receive ADT supplemented by local therapy^7^, the optimal extent of systemic treatment is still being evaluated. Selected cN1 patients presenting with additional risk factors might benefit from a combination of standard ADT with novel hormonal therapy^8^, whereas survival advantage in others remains unclear^9^. In described setting identification of candidates for extended staging as well as defining pN1 individuals with a poor prognosis has become of significant value. A cross-talk of immune cells in a metastatic node has attributed the novel haematological markers (neutrophil-to-lymphocyte ratio [NLR], platelet-to-lymphocyte ratio [PLR], systemic immune–inflammation index [SII] and neutrophil-to-erythrocyte [NER]) not only with biological but also with clinical value^10–13^.

This study aimed to determine the association of novel haematological markers with PCa nodal involvement as well as evaluate their prognostic value in patients with pN1.

## Material and methods

### Patients

The study was conducted following the guidelines of the Declaration of Helsinki and approved by the Ethics Committee of the Medical University of Warsaw (nr AKBE/58/2022; 21 February 2022). Informed consent was obtained from all subjects involved in the study.

This observational study comprised patients with clinically nonmetastatic PCa treated with RP and eLND from 2012 to 2018 in a single tertiary centre. We included patients with intermediate to high-risk PCa (according to the European Association of Urology risk groups) only. Previous radio- or hormonotherapy constituted exclusion criteria. In patients from the intermediate group decision on lymphadenectomy was at the physician's discretion and was based mainly on preoperative nomograms^2,3^.

### Data collection

Retrospective clinical and pathological data were collected from the prospective department database. Blood cell counts used for further analysis were obtained from routinely performed preoperative evaluations. The neutrophil-to-lymphocyte ratio (NLR), platelet-to-lymphocyte ratio (PLR), systemic immune–inflammation index (SII) and neutrophil-to-erythrocyte (NER) were calculated as reported previously^11,14^. The Central Statistical Office, Poland's main government agency in charge of statistics and census data, including a full registry of deaths, was consulted for survival follow-up information.

### Statistical analysis

Analyses were performed using SAS 9.4 software (SAS Institute, Cary, NC, USA). Qualitative and continuous variables were compared using Fisher’s exact test and Mann–Whitney’s U-test, respectively. To avoid overfitting median value was utilized to obtain categorized variables when constructing multivariate models as well as comparing survival outcomes. Cut-off aimed at excluding nodal involvement with minimal false negatives was optimized to provide maximal negative predictive value (NPV). Overall survival estimates were calculated using the Kaplan–Meier method supplemented with the log-rank test. The threshold for significance was set at *p* < 0.05.

### Informed consent

Informed consent was obtained from all individual participants included in the study.

### Ethics approval

The study was approved by the Ethics Committee of the Medical University of Warsaw (nr AKBE/58/2022; 21 February 2022).

## Results

### Baseline characteristics of the study cohort

**Figure 1:**
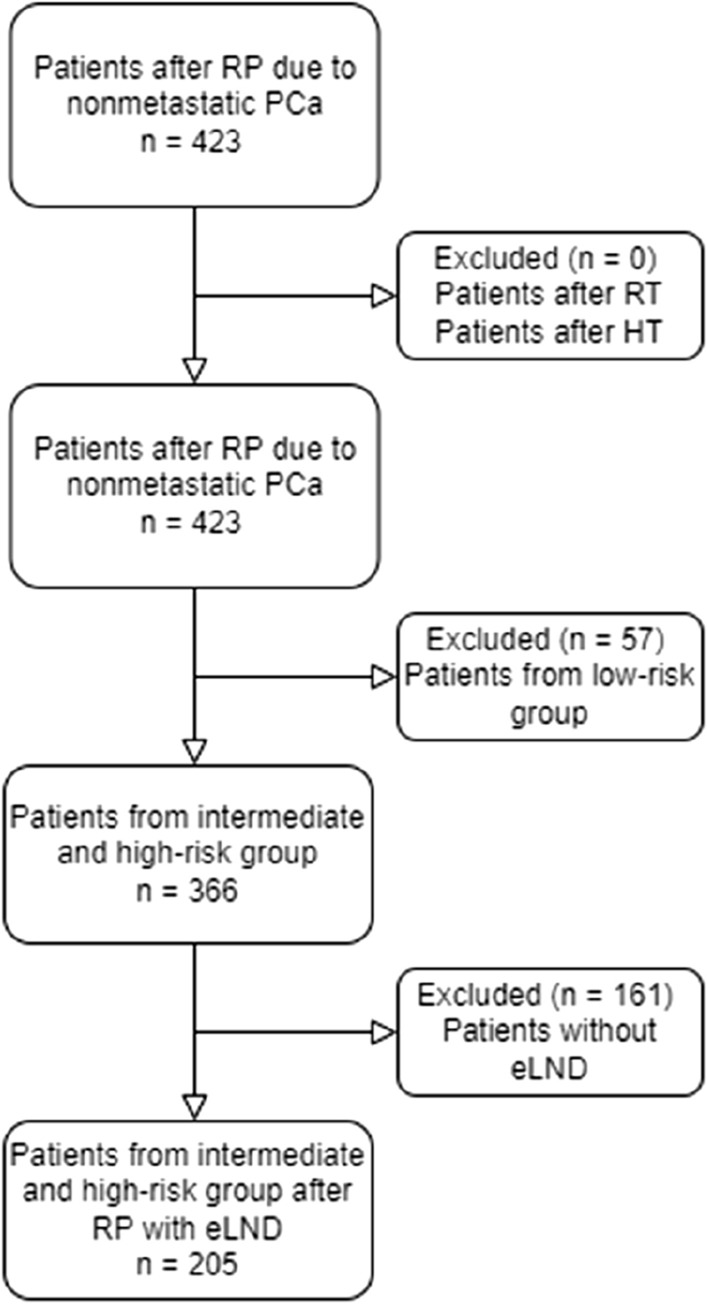
Inclusion and exclusion criteria—flow diagram. Graphics were printed using https://app.diagrams.net/.

Out of 423 patients treated for nonmetastatic prostate cancer in the analyzed period, a total of 205 patients fulfilled the inclusion criteria (Fig. 1). Baseline characteristics stratified by pathologic nodal staging are summarized in Table 1.

**Table 1:** Baseline characteristics of patients stratified by nodal status in the postprostatectomy specimen.

Variable	Overall	pN0 n = 174 (84.88%)	pN1 n = 31 (15.12%)	*p*
PSA (ng/mL, mean/IQR)	19.05/9.5	17.26/8.8	29.23/16.20	0.0015
**cT (n,%)**				
cT1	52 (26.40%)	46 (27.88%)	6 (19.35%)	0.18
cT2	140 (71.07%)	116 (70.30%)	23 (74.19%)	
≥ cT3	5 (2.54%)	3 (1.82%)	2 (6.45%)	
**Biopsy ISUP grade group (n, %)**				
1	41 (20.10%)	35 (20.35%)	5 (16.13%)	0.0123
2	62 (30.39%)	58 (33.72%)	4 (12.90%)	
3	40 (19.61%)	33 (19.19%)	7 (22.58%)	
4	41 (20.10%)	34 (19.77%)	7 (22.58%)	
5	20 (9.80%)	12 (6.98%)	8 (25.81%)	
NLR (mean/IQR)	3.66/1.92	3.86/1.85	2.53/1.22	0.0025
NER (mean/IQR)	1.23/0.53	1.26/0.57	1.01/0.38	0.0568
PLR (mean/IQR)	134.13/59.31	136.28/59.22	123.42/53.60	0.07
SII (mean/IQR)	760.81/436.36	791.65/454.95	586.42/347.53	0.0047
Age (years, mean/IQR)	65/7	65/8	64/5	0.34
**Prostatectomy ISUP grade group (n, %)**				
1	13 (6.34%)	11 (6.32%)	2 (6.45%)	< 0.0001
2	58 (28.29%)	58 (33.33%)	0 (0%)	
3	49 (23.90%)	45 (25.86%)	4 (12.90)	
4	48 (23.41%)	36 (20.69%)	12 (38.71%)	
5	37 (18.05%)	24 (13.79%)	13 (41.94%)	
**pT (n, %)**				
pT2	91 (44.39%)	87 (50%)	4 (12.90%)	< 0.0001
pT3	112 (54.63%)	87 (50%)	25 (80.65%)	
pT4	2 (0.98%)	0	2 (6.45%)	
EPE (n,%)	114 (55.61%)	87 (50%)	27 (87.10%)	< 0.0001
SVI (n,%)	51 (24.76%)	30 (17.24%)	21 (67.74%)	< 0.0001
PSM (n,%)	78 (38.06%)	58 (33.33%)	20 (64.52%)	0.0021
LRP (n,%)	35 (17.00%)	32 (18.39%)	3 (9.68%)	0.30

*cT* clinical staging, *EPE* extracapsular extension, *IQR* interquartile range, *LRP* laparoscopic radical prostatectomy, *NER* neutrophil-to-erythrocyte, *NLR* neutrophil-to-lymphocyte ratio, *PLR* platelet-to-lymphocyte ratio, *pN* pathological nodal staging, *PSA* prostate-specific antigen (ng/mL), *PSM* positive surgical margins, *pT* pathological local staging, *SII* systemic immune-inflammation index, *SVI* seminal vesicles involvement.

### Association of haematological markers and nodal involvement

Patients with pN1 had significantly lower NLR and SII in univariate analysis. For NER and PLR there was some evidence which did not meet a conventional level of statistical significance (Table 1). Univariate continuous pre-prostatectomy variables were then categorized and used to develop multivariable models. In multivariable analysis, categorized NLR (NLR ≥ 2.7 OR 0.38; 95% CI 0.15–0.94) constituted a predictor of nodal involvement independently from biopsy grading and PSA (Table 2) with the c-index reaching 0.80. We have also observed that patients with nodal involvement presented significantly higher lymphocytes count (2.06 × 10^3^ vs 1.83 × 10^3^; *p* = 0.034) but insignificantly lower neutrophil count (4.50 × 10^3^ vs 5.56 × 10^3^; *p* = 0.054), indicating that lymphocytes were primarily responsible for the NLR decline.

**Table 2:** The association of haematological markers and nodal involvement—multivariable logistic regression analysis.

Variable	OR (95% CI)	*p*
**NLR model (AUC** **=** **0.795)**		
NLR ≥ 2.7	0.38 (0.15–0.94)	0.0367
**ISUP grade group**		
1	1	0.0383
2	0.64 (0.13–3.03)	
3	1.96 (0.53–7.26)	
4	2.03 (0.54–7.54)	
5	6.11 (1.51–24.76)	
**PSA**		
< 10 ng/mL	1	0.014
10–20 ng/mL	3.24 (1.15–9.13)	
≥ 20 ng/mL	4.76 (1.57–14.38)	
**NER model (AUC** **=** **0.776)**		
NER ≥ 0.98	0.91 (0.38–2.16)	0.83
**ISUP grade group**		
1	1	0.0242
2	0.63 (0.13–2.93)	
3	1.95 (0.54–7.05)	
4	2.12 (0.57–7.93)	
5	6.45 (1.63–25.54)	
**PSA**		
< 10 ng/mL	1	0.0076
10–20 ng/mL	3.51 (1.26–9.76)	
≥ 20 ng/mL	5.17 (1.73–15.42)	
**SII model (AUC** = **0.785)**		
SII ≥ 575	0.44 (0.18–1.01)	0.0783
**ISUP grade group**		
1		0.0348
2	0.54 (0.11–2.56)	
3	1.73 (0.47–6.38)	
4	1.84 (0.50–6.81)	
5	5.59 (1.40–22.32)	
**PSA**		
< 10 ng/mL	1	0.0141
10–20 ng/mL	3.04 (1.08–8.61)	
≥ 20 ng/mL	4.86 (1.62–14.63)	
**PLR model (AUC** = **0.774)**		
PLR120 ≥ 2.7	0.58 (0.24–1.39)	0.2179
**ISUP grade group**		
1	1	0.0363
2	0.63 (0.13–2.98)	
3	1.86 (0.51–6.81)	
4	2.37 (0.64–8.77)	
5	5.89 (1.46–23.74)	
**PSA**		
< 10 ng/mL	1	0.0069
10–20 ng/mL	3.37 (1.20–9.44)	
≥ 20 ng/mL	5.45 (1.82–16.35)	

*OR* odds ratio, *CI* confidence interval, *NLR* neutrophil-to-lymphocyte ratio, *PSA* prostate-specific antigen, *NER* neutrophil-to-erythrocyte, *SII* systemic immune-inflammation index, *PLR* platelet-to-lymphocyte ratio.

### Haematological markers in patients presenting extraprostatic extension, seminal vesicle involvement and high-grade prostate cancer

There was no significant difference in NLR between patients with and without EPE (3.44 vs 3.93, *p* = 0.2385). NLR has also not differed significantly between patients presenting SVI and those without it (3.36 vs 3.76, *p* = 0.4629).

Additionally, there was no statistical difference regarding NER (1.22 vs 1.21, *p* = 0.9549), PLR (127.27 vs 143.35, *p* = 0.0730) or SII (705.26 vs 830.82,* p* = 0.0898) between patients with and without EPE. Finally, there were no significant differences between patients with and without SVI for NER (1.26 vs 1.21, *p* = 0.779), PLR (130.26 vs135.42, *p* = 0.8021) or SII (738.33 vs768.30, *p* = 0.525).

Patients with high-grade PCa (ISUP IV or V) showed a non-significantly lower NLR than those with ISUP III or less (3.27 vs. 3.93, *p* = 0.1426). The differences between NER (1.19 vs 1.23, *p* = 0.5759), PLR (130.38 vs 137.15, *p* = 0.5016) and SII (701.94 vs 802.36, *p* = 0.4291) were also not statistically significant.

### Validation of NLR as a marker of nodal involvement

To introduce NLR as a stratification tool when aiding the decision on lymphadenectomy we tested different cut-offs to select the threshold offering maximal NPV with satisfactory positive predictive value (PPV) (Table 3) and validated them internally using utilizing bootstrapped dataset (n = 200). The most optimal cut-off set at 4.1 would spare 38 (22.09%) out of 172 unnecessary lymphadenectomies missing 2 patients (6.45%) out of 31 bearing pN1.

**Table 3:** Different NLR cut-offs validated internally using the bootstrapped dataset (n = 200).

NLR cut-off sparing eLND	NPV (95% CI)*	PPV (95% CI)*	pN0 eLND spared (%)	pN1 missed (%)
≥ 3	91.13% (85.38–97.41%)	20.35% (13.82–27.66%)	44.19	19.35
≥ 4	91.18% (83.33–98.09%)	17.66% (12.3–23.91%)	23.84	9.68
≥ 4.1	92.66% (84.91–100%)	17.82% (12.24–23.87%)	22.09	6.45
≥ 4.5	91.63 (83.29–100%)	17.28% (11.87–23.08%)	19.19	6.45
≥ 5	90.20 (80–100%)	16.77% (11.5–22.48%)	16.28	6.45

*NLR* neutrophil-to-lymphocyte ratio, *eLND* extended lymphadenectomy, *NPV* negative predictive value, *PPV* positive predictive value.

*Values calculated for bootstrapped dataset (n = 200 × 205).

### If present, high NLR is a poor survival prognosticator in pN1

**Figure 2:**
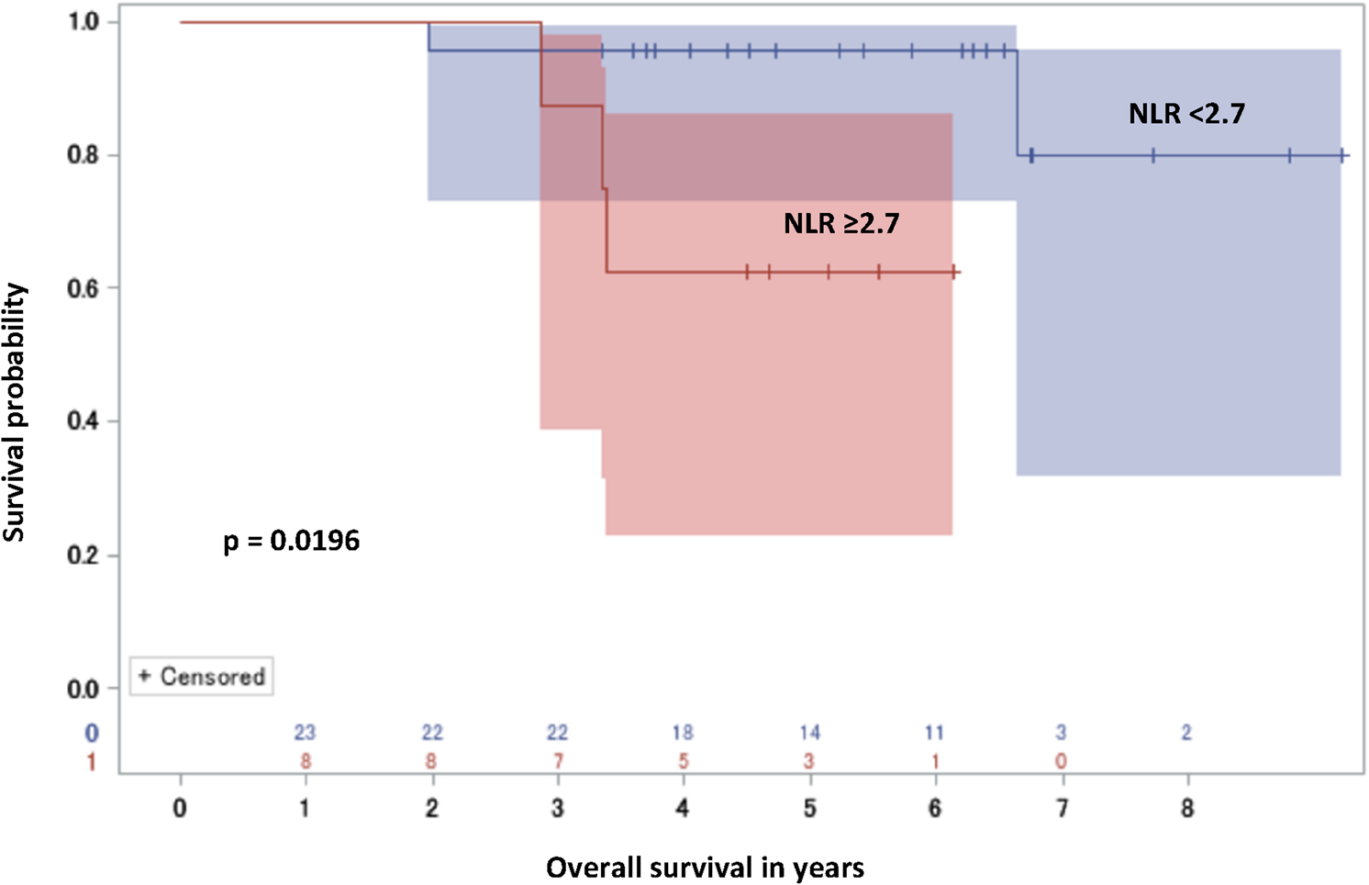
Overall survival for pN1 patients stratified with NLR (≥ 2.7 vs < 2.7). Graphics were printed using SAS software.

Patients with nodal involvement were followed up for a median of 72.2 months (95%CI 58.9–82). A total of 2/23 (8.7%) and 3/8 (37.5%) patients with low NLR (< 2.7) and high NLR (≥ 2.7) died during follow-up, respectively. Kaplan–Meier analysis of patients bearing pN1 indicated that individuals with high NLR achieved significantly shorter overall survival (Fig. 2) (*p* = 0.0196).

## Discussion

In this study, we demonstrate the equivocal value of the neutrophil-to-lymphocyte ratio in patients treated with radical prostatectomy and extended lymphadenectomy. Contrary to the previous studies^11,12,15–20^, we have found that high NLR might be linked with a decreased risk of nodal involvement. On the other hand, we discovered that in patients with pN + elevated NLR was a predictor of poor survival.

In the majority of the previous studies involving patients with solid neoplasms, high NLR was associated with an increased risk of nodal involvement which is the opposite of our findings^12,15–19^. On the other hand, previous studies investigating the impact of haematological markers on NI exclusively in prostate cancer have produced conflicting results. Analysis of a large cohort of 1367 PCa patients after RP by Lee et al. failed to confirm NLR as a marker of nodal invasion^21^. Similarly, a modest cohort analysed by Maeda et al. has revealed an insignificant impact of NLR on adverse pathological features including positive nodal status^22^. On the other hand, the study by Özsoy et al. yielded positive validation of NLR ≥ 3 as predictive of all pathological features including nodal involvement which translated into a higher risk of BCR. The underlying clinical relevance of the index may therefore be attributable to its connection with unfavourable pathological characteristics (APF), as the NLR failed to continue to be an independent predictor of BCR in multivariate analysis^20^. The systemic immune-inflammation index (SII) which is a derivate of NLR multiplied by platelet count has also been linked with nodal involvement^11^. In the study by Rajwa et al. prevalence of pN1 in patients presenting high SII was 3.4% whereas in patients with low SII positive node status was confirmed in 1.4% of cases. In the study by Lu et al.^23^ Authors found no correlation between NLR and nodal status. Surprisingly, patients with adverse local staging (pT3-4) presented significantly lower NLR which is inverse to results observed previously, but in line with our findings. Finally, a recent study on 1258 patients by Bravi et al. found that patients with higher NLR were at lower risk of nodal involvement (OR: 0.77; *p* = 0.005), which is also opposite to the previously published data. NLR > 2 was linked to a 10% risk of NI when probability splines were used and the cancer severity was taken into account. Patients with lower NLR had an increased risk of NI, which translated into an increased risk of BCR but only in the unstratified cohort and the univariate analysis.

Nevertheless, the study by Bravi et al. was the first to break the existing rule that high NLR always predicts a negative end-point. To explain the discrepancy with previous studies Authors pustulated differences in the risk profile of the groups, the extent of lymphadenectomy, dietary habits, pN1 prevalence and age^13^. These explanations appear to apply to our cohort as well. In studies presenting high rates of pN1 in patients with elevated haematological markers, the overall prevalence of nodal involvement was significantly lower (1.9–9.4%)^10,20–24^ when compared to the cohort presented in our study and the one presented by Italian authors (15% and 17%, respectively)^13^. Following variations in pN1 rates might implicate different selections for lymphadenectomy which might affect NLR as an NI prognosticator. Firstly, the parametric evaluation of NLR is strongly dependent on the cohort analyzed. Mean and median values have differed significantly among the studies performed to date^10,20–24^, which might have several implications. In departments performing eLND in all patients including individuals from the low-risk group^11,20,21,23^, NLR might range significantly wider. For instance, the cohort by Özsoy included 699 patients bearing pN1 (9.4%) with an overall rate of 23% of patients presenting NLR ≥ 3^20^. Since this quartile cut-off value is close to the median NLR value in our study, it is reasonable to assume that the NLR values provided by our patients were much higher. In subgroup analysis in the same study high NLR was associated with 2.1 OR when predicting adverse pathology in the low-risk group, but with only 1.7 OR in the intermediate group, while NLR ≥ 3 was not significantly correlated with either pT3 or NI in the high-risk group. This not only suggests a non-linear association between NLR and NI but also implies a significant reliance on the cohort's risk profile. A significant part of our cohort was treated in the pre-MRI era with local staging limited to DRE and nodal staging limited to CT which might contribute to understaging and increased prevalence of pN1. On the other hand, in departments with very low pN1 rates, some patients bearing pN1 might be understaged as a result of poor lymphadenectomy selection or undersampling. In this scenario “true” pN1 individuals hidden under pNx diagnoses are excluded from the final risk assessment. Consequently, the true range of NLR presented by patients with nodal involvement might be falsely narrowed. For instance, the study by Lee et al. involved only 30 (2.2%) patients with NI, setting the cut-off defining “high” NLR at the level of 2.5^21^. The exact number of pN1 patients with NLR between 2.5 and 4 has not been presented in this study. Finally, another potential confounder is age. The median age in our group was the same as reported by Bravi (65 years) whereas in previous studies it ranged from 59 to 61^11,20,22,25^. Since NLR has been shown to increase with age^26^, this difference might also explain discrepancies in the association between NLR and NI.

On the basis of prior hypotheses, NLR can be interpreted as a manifestation of systemic inflammation, a reflection of immune system efficiency and relative changes between immune cell subpopulations in response to tumour spread. We assume that in patients that are at high baseline risk of bearing nodal involvement (intermediate and high-risk group) low NLR might reflect lymphocyte count increase as a systemic reaction to cancerous cells. Therefore, low NLR constitutes a potential marker of NI in intermediate/high-risk prostate cancer patients. Simultaneously, in patients that are already confirmed as pN1 high NLR can be attributed to immune exhaustion, systemic inflammation associated with comorbidities and poor anti-tumour reaction. Therefore in these patients, high NLR constitutes a marker of poor survival prognosis, which seems to complement outcomes of previous studies linking NLR with biochemical recurrence^11,20,21,27^.

Our study has several limitations. Since this is an observational study with retrospective data collection selection bias cannot be ruled out. To control confounding we have restricted enrollment and performed statistical control of potential confounders. However, due to variable eLND qualification in intermediate-risk patients and lack of standardization of eLND extent, our outcomes might slightly differ from analogous cohorts. The inability to obtain data on post-RP adjuvant and salvage therapy made it difficult to identify and assess bias affecting follow-up. When analysing pN1 patients' follow-up we aimed at evaluating overall survival, however, the lack of cancer-specific follow-up data prevented us from defining the contribution of cancer-dependent mortality.

In conclusion, to the best of our knowledge, our study is the first to report the ambiguous value of preoperative NLR in predicting NI in preprostatectomy setting and overall survival in pN1 patients in postprostatecomy setting. We have validated internally NLR as a supplement to PSA and biopsy grading when predicting nodal status and evaluated the safety of implementing NLR in excluding NI. We believe different outcomes of NLR validation in postprostatectomy cohorts to date require further research with genuine subgroup analysis and selection bias control. With consecutive validation, NLR might be utilized in both pre- and postprostatectomy models.

## Data Availability

The data sets used and analyzed during the current study are available from the corresponding author on a reasonable request.
